# Predicting Flow Rate Escalation for Pediatric Patients on High Flow Nasal Cannula Using Machine Learning

**DOI:** 10.3389/fped.2021.734753

**Published:** 2021-11-08

**Authors:** Joshua A. Krachman, Jessica A. Patricoski, Christopher T. Le, Jina Park, Ruijing Zhang, Kirby D. Gong, Indranuj Gangan, Raimond L. Winslow, Joseph L. Greenstein, James Fackler, Anthony A. Sochet, Jules P. Bergmann

**Affiliations:** ^1^Department of Biomedical Engineering, Institute for Computational Medicine, Johns Hopkins University, Baltimore, MD, United States; ^2^Division of Health Sciences Informatics, Johns Hopkins University School of Medicine, Baltimore, MD, United States; ^3^Department of Anesthesiology and Critical Care Medicine, Johns Hopkins University School of Medicine, Baltimore, MD, United States; ^4^Division of Pediatric Critical Care Medicine, Department of Pediatrics, Johns Hopkins All Children's Hospital, St Petersburg, FL, United States

**Keywords:** high flow nasal cannula, flow rate escalation, pediatric critical care, non-response, machine learning, acute respiratory failure

## Abstract

**Background:** High flow nasal cannula (HFNC) is commonly used as non-invasive respiratory support in critically ill children. There are limited data to inform consensus on optimal device parameters, determinants of successful patient response, and indications for escalation of support. Clinical scores, such as the respiratory rate-oxygenation (ROX) index, have been described as a means to predict HFNC non-response, but are limited to evaluating for escalations to invasive mechanical ventilation (MV). In the presence of apparent HFNC non-response, a clinician may choose to increase the HFNC flow rate to hypothetically prevent further respiratory deterioration, transition to an alternative non-invasive interface, or intubation for MV. To date, no models have been assessed to predict subsequent escalations of HFNC flow rates after HFNC initiation.

**Objective:** To evaluate the abilities of tree-based machine learning algorithms to predict HFNC flow rate escalations.

**Methods:** We performed a retrospective, cohort study assessing children admitted for acute respiratory failure under 24 months of age placed on HFNC in the Johns Hopkins Children's Center pediatric intensive care unit from January 2019 through January 2020. We excluded encounters with gaps in recorded clinical data, encounters in which MV treatment occurred prior to HFNC, and cases electively intubated in the operating room. The primary study outcome was discriminatory capacity of generated machine learning algorithms to predict HFNC flow rate escalations as compared to each other and ROX indices using area under the receiver operating characteristic (AUROC) analyses. In an exploratory fashion, model feature importance rankings were assessed by comparing Shapley values.

**Results:** Our gradient boosting model with a time window of 8 h and lead time of 1 h before HFNC flow rate escalation achieved an AUROC with a 95% confidence interval of 0.810 ± 0.003. In comparison, the ROX index achieved an AUROC of 0.525 ± 0.000.

**Conclusion:** In this single-center, retrospective cohort study assessing children under 24 months of age receiving HFNC for acute respiratory failure, tree-based machine learning models outperformed the ROX index in predicting subsequent flow rate escalations. Further validation studies are needed to ensure generalizability for bedside application.

## Introduction

Acute respiratory failure is one of the most common indications for hospitalization among pediatric patients (1). For children under 5 years old, it makes up 2% of mortalities in the United States and 18% worldwide (2). In severe cases where children experience acute respiratory failure, physicians trial a variety of non-invasive ventilation (NIV) modalities such as heated, humidified, high flow nasal cannula (HFNC) to improve tissue oxygenation, decrease patient work of breathing, and limit exposure to invasive mechanical ventilation (MV) (3). While at times necessary, intubation and MV place children at risk for acquired complications and require higher levels of monitoring, skilled personnel, and supportive resources located in a pediatric intensive care unit (PICU) setting (4).

Over the past twenty-five years, HFNC use for acute respiratory failure has increased in popularity, as evident in several epidemiologic assessments that also reveal a concurrent chronologic tapering of MV rates (5–9). Patients that escalate to a ventilator after a trial of HFNC are thought to have experienced HFNC non-response. Although multiple clinical characteristics have been identified as potential risk factors for HFNC non-response, such as comorbid cardiac disease, hypercarbia, or persistent tachypnea and tachycardia, the prediction of HFNC non-response remains challenging due to study heterogeneity, regional practice variation, lack of clarity in the indications for device escalation, and subjective definitions of non-response (10–17). Simple clinical metrics have been developed using vital sign data, such as the respiratory rate-oxygenation (ROX) and ROX-heart rate (ROX-HR) indices, to predict MV after HFNC initiation (18, 19). For adults, Roca et al. found within 12 h of scoring, the ROX index yields an area under the receiver operating characteristic (AUROC) of 0.76 (18). A limitation of the ROX, and other indices that use the ratio of oxygen saturation (S_p_O_2_) to inspired oxygen fraction (F_i_O_2_), is that as S_p_O_2_ increases, the measurement progressively loses sensitivity to changes in F_i_O_2_. When S_p_O_2_ ≥ 97%, failure to appropriately wean F_i_O_2_ can significantly bias the ROX score.

At present, there are no guidelines for determining the level of respiratory support required for patients with acute respiratory failure. After initiating HFNC, flow rates are adjusted using clinical judgment with subjective determinations of patient stability and response to therapy (20, 21). Machine learning techniques may allow for more robust and precise metrics to not only predict HFNC non-response but also inform ideal settings and alert providers prior to impending respiratory failure. We hypothesize machine learning techniques using a combination of patient demographics, HFNC settings, medications, vital sign indices, and medical history can be used to develop a predictive model for HFNC flow rate escalation.

## Methods

### Study Design

We assessed a retrospective cohort of children <2 years old admitted to the Johns Hopkins Children's Center Pediatric Intensive Care Unit (PICU) between January 2019 through October 2020 for acute respiratory failure and placed on HFNC. We excluded patients in which MV occurred prior to HFNC and those who were electively intubated in the operating room (OR). This study was reviewed and approved by the Johns Hopkins Medicine Institutional Review Board (IRB#00211399).

### Data Source

We identified patients by automated query of electronic health record (EHR) demographics and respiratory support documentation for all PICU admissions during the study period. Demographics, vital signs, nursing/respiratory care observations, medication administrations, and medical history were extracted from the EHR for all included patient encounters. Patient encounters were split into training and testing sets at a 60/40 ratio. Table 1 shows descriptive analyses for comparing cohorts with and without escalation using demographic data, comorbidities, and starting flow rate parameters.

**Table 1:** Descriptive characteristics for the study population, including demographics, anthropometrics, and comorbidities, stratified by the presence of one or more flow rate escalations.

**Variables**	**Total (*N* = 433)**	**Escalation Encounters (*N* = 335)**	**Non-Escalation Encounters (*N* = 98)**	***P*-Value**
**Age [months]**				
Median (IQR)	6.0 (2.0–14.0)	7.0 (2.0–14.0)	4.5 (2.0–11.8)	0.056
**Weight [kg]^a^**				
Median (IQR)	7.2 (4.9–9.4)	7.3 (4.9–9.4)	6.5 (4.9–8.7)	0.439
**Sex (%)**				0.809
Male	241 (55.7)	188 (56.1)	53 (54.1)	
Female	192 (44.3)	147 (43.9)	45 (45.9)	
**Ethnicity (%)**				0.733
Hispanic	51 (11.8)	38 (11.3)	13 (13.3)	
Non-Hispanic	382 (88.2)	297 (88.7)	85 (86.7)	
Race (%)^*^				0.046
White	190 (43.9)	135 (40.3)	55 (56.1)	
Black	155 (35.8)	130 (38.8)	23 (23.5)	
Asian	18 (4.2)	13 (3.9)	4 (4.1)	
Other	77 (17.9)	56 (16.7)	16 (16.3)	
Declined	1 (0.2)	1 (0.3)	0 (0.0)	
Bacterial Pneumonia Treatment (%)^b^^^*^^				0.008
No Treatment Present	406 (93.8)	308 (91.9)	98 (100.0)	
Treatment Present	27 (6.2)	27 (8.1)	0 (0.0)	
Mechanically Ventilated (%)^*^	20 (4.6)	20 (6.0)	0 (0.0)	<0.001
**Number of Flow Rate Escalations**				
Median (IQR)^*^	0.4 (0.1–0.7)	0.5 (0.3–0.7)	—	<0.001
**Duration of HFNC (hrs.)**				
Median (IQR)^*^	36.2 (17.9–61.3)	42.0 (22.4–70.2)	19.5 (10.7–36.2)	<0.001
**Starting Flow Rate [L/min]**				
Mean ± SD	5.6 ± 4.0	5.2 ± 4.3	6.8 ± 2.3	
Median (IQR)^*^	6.0 (3.0–8.0)	6.0 (2.0–8.0)	6.0 (5.2–8.0)	<0.001
**Starting Flow Rate by Weight [L/kg/min]^a^**				
Mean ± SD	0.8 ± 0.6	0.8 ± 0.6	1.1 ± 0.5	
Median (IQR)^*^	0.8 (0.4–1.2)	0.8 (0.3–1.1)	1.0 (0.7–1.4)	<0.001
**Maximum Flow Rate [L/min]**				
Median (IQR)^*^	8.0 (6.0–10.0)	8.0 (7.0–10.0)	6.0 (5.2–8.0)	<0.001
**Maximum Flow Rate by Weight [L/kg/min]^a^**				
Median (IQR)^*^	1.2 (0.9–1.7)	1.3 (1.0–1.8)	1.0 (0.7–1.4)	<0.001
**Starting F**_**i**_**O**_**2**_ **[%]**				
Median (IQR)	40.0 (30.0–60.0)	40.0 (21.0–60.0)	40.0 (30.0–50.0)	0.448
**Length of Stay [hrs.]**				
Median (IQR)^*^	87.7 (61.8–148.5)	102.3 (69.3–180.3)	60.9 (41.9–82.9)	<0.001

a *For descriptive purposes only, these values represent analysis on 395/433 (306 escalated, 89 non-escalated) patients that had weights recorded*.

b *Bacterial pneumonia diagnoses were established by usage of antibiotics during hospital stay for > 5 days*.

* *p-value < 0.05*.

### Model Outcome, Time Window, and Lead Time

**Figure 1:**
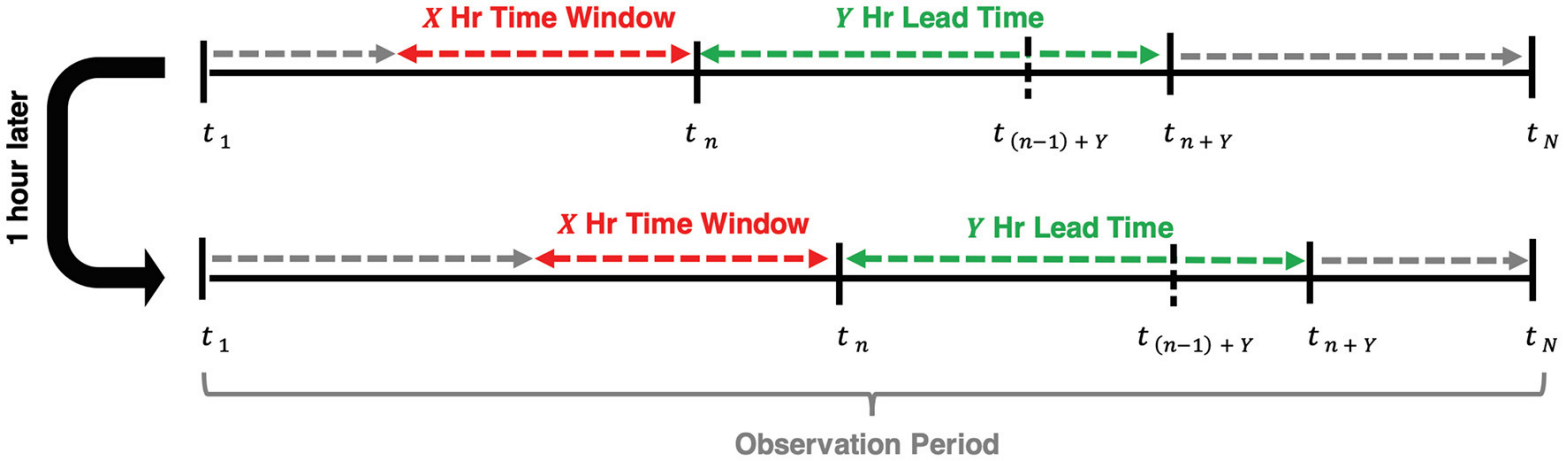
Visual representation of the relationship between time window, lead time, and the time escalation is predicted at each hour, t_n_, of admission (*n* = 1,2,…,N). The observation period begins at the time of admission and concludes at t_N_ when the patient escalates to MV for ventilated patients or at discharge for non-ventilated patients.

Figure 1 shows a simple example that depicts the concepts of the observation period, time window, and lead time for a single patient encounter. To incorporate time-series features like HFNC settings and vitals data, we defined the observation period for a patient encounter as the interval of time over which data is collected and aggregated to generate a prediction. The observation period ends either at the time a patient was escalated to a ventilator or at date and time of hospital discharge for those who never received MV. The observation period is segmented into shorter overlapping time intervals called time windows with constant duration and start times that occur every 1 h until the end of an observation period. We varied the length of time windows to be 2, 4, 8, and 12 h. We evaluated models using lead times of 1, 2, 6, and 12 h.

Our primary model outcome, flow rate escalation, is defined as follows. We define n (*n* = 1, 2, 3, …, N) as an index to each of the N flow rate predictions made for a patient and define t_n_ as the time of the n^th^ prediction. A prediction of flow rate was made (and n incremented by 1) every hour of a patient's observation period. We predicted a flow rate at a lead time of Y hours ahead of t_n_ (i.e., at time t_n_ + Y) using data collected across a time window beginning at X hours in the past (i.e., the time window extending from t_n_-X to t_n_). A prediction of flow rate escalation was defined to occur when the predicted flow rate at t_n_+ Y was greater than that predicted at t_n−1_+ Y.

### Outlier Rejection

Prior to feature extraction, vital sign values outside a plausible physiologic range were removed. Ranges were established from multicenter cross-sectional studies on hospitalized pediatric patients and the consensus of investigators (22–24). The excluded values represent data entries that were noisy, misinterpreted, missing, measured at the wrong time, or the result of keystroke errors (22). Specifically, these ranges were heart rates outside 5–300 beats per min, respiratory rates outside 5–120 breaths per min, temperatures outside 70–110 degrees Fahrenheit, and S_p_O_2_ outside 60–100 percent. Mean arterial pressure (MAP) outliers were defined by values higher than the maximum systolic blood pressure or lower than the minimum diastolic blood pressure for the entire cohort of patients as MAP is a weighted average between the systolic and diastolic blood pressures. Of the 424,343 available recordings from unique encounters, 7,128 (1.7%) were considered outliers and removed from the dataset.

### Feature Extraction and Missing Value Imputation

At each prediction time, we considered a combination of static and dynamic features. Static features include patient demographics and medical history for each encounter. Demographics data were one-hot encoded from the information available at the time of admission for a given encounter and treated as static throughout hospitalization. Medical history features consisted of whether a patient had any chronic diagnoses, active problems, or principal diagnosis at the time of admission.

Dynamic features included validated vital signs (e.g., heart rate, blood pressure, respiratory rate, and S_p_O_2_), F_i_O_2_, flow rate, medication administrations, and synthetic features (described in the next section). Supplementary Table 1 shows a complete list of features. If one or more samples were available in the time window, summary statistics of minimum, maximum, median, mean, delta (change in value from start to end of the time window), standard deviation, and the number of times observed for each feature were computed. If no sample was available in the window, the last available value was used, known as forward fill. Forward fill was selected as an imputation strategy for vitals data because the objective was to predict a clinical decision. Forward fill simulates a clinician's perception regarding the clinical status of a patient using previously observed but currently unknown vital signs. If no previous value was available for F_i_O_2_ or flow rate, the average initiation value of the patient cohort was used, which is also known as mean-value substitution. Supplementary Table 2 shows the average values for each level of respiratory support.

Medication administrations were one-hot encoded to indicate whether the specific medication was administered or not. Once a medication was given, a value of one was added to the cumulative total number of administrations for the appropriate antibiotic or pharmaceutical class. Unlike other observations, the number of administrations did not reset at the start of each time window but instead carried over its current count from one window to the next. Medications synonymous with intubation, such as neuromuscular blocking agents, were discounted as potential features, as their administration is determined from a clinician already deciding the need for future MV and would bias a flow rate escalation prediction.

### Synthetic Features

**Figure 2:**
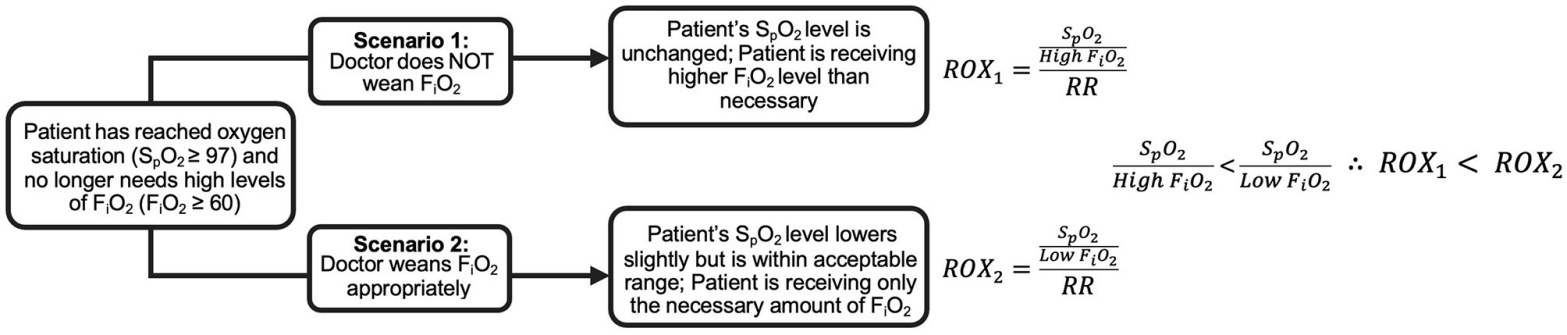
Comparison of ROX index for a hypothetical patient with S_p_O_2_ ≥ 97% given two different clinician approaches. In Scenario 1, the clinician does not decrease the patient's F_i_O_2_, resulting in a low ROX index. In Scenario 2, the clinician decreases the patient's F_i_O_2_ appropriately, resulting in a higher ROX index than Scenario 1, despite the patient's identical health status.

Synthetic features combining raw features were also used for prediction. To evaluate the usefulness of the ROX and ROX-HR indices, we included them as features. The ROX index is defined as the ratio of S_p_O_2_ to F_i_O_2_ divided by respiratory rate (RR). The ROX-HR index is the ROX index divided by HR. We calculated the ROX and ROX-HR as the average score within each time window. Because of the sigmoid shape of the oxygen-hemoglobin dissociation curve, the change in S_p_O_2_ relative to a change in F_i_O_2_ decreases as S_p_O_2_ increases. Above 97%, S_p_O_2_ loses sensitivity to changes in F_i_O_2_ (25). Figure 2 shows how this can bias the ROX score if clinicians are slow to decrease F_i_O_2_ when S_p_O_2_ is high (26). To account for this, we included a one-hot oversaturation variable set to 1 if F_i_O_2_ ≥ 60% despite S_p_O_2_ ≥ 97%. Finally, we included the number of times the F_i_O_2_ was adjusted within the time window as a feature.

### Binary Classification Models

We trained random forest, logistic regression, and gradient boosting classifiers using the full feature set from the training data with five-fold cross-validation for model selection. Supplementary Figures 3–6 show that gradient boosting models outperform both random forest and logistic regression models for all of our time window and lead time combinations. Therefore, all model results will explore the performance of gradient boosting.

### Evaluation Metrics

For each machine learning algorithm, we evaluated time windows of 2, 4, 8, and 12 h and lead times of 1, 2, 6, and 12 h. Model performance, which was measured with AUROC and area under the precision-recall curve (AUPRC), was evaluated only on the withheld testing set which consisted of 40% of encounters excluded from model training. To compare performance against the ROX and ROX-HR baselines, we built models using only ROX and ROX-HR as features. To distinguish performance difference, we compared the performance of the logistic regression ROX/ROX-HR models against our best full-feature model.

We evaluated feature importance using Shapley additive explanations (27). A Shapley value describes the difference in model performance given the inclusion and exclusion of a specific feature. Shapley feature importance values were calculated for all features in the generated model by using the Python Shap3 package (28). Any features that were determined to have a Shapley value of 0, indicating no effect on our model's performance, were eliminated.

For the descriptive analysis, categorical comparisons were established using a chi-squared test, continuous variables were compared using Welch's *t*-test, and medians of continuous variables were compared using a Kruskal-Wallis test using the python package TableOne (29).

### Institutional HFNC Practice

After initial treatment with nasopharyngeal suctioning and low-flow nasal cannula (NC), patients with persistent respiratory distress were transitioned to HFNC with a flow rate between 4 and 15 L/min, based on clinician assessment of patient work of breathing, and F_i_O_2_ between 40 and 100% (adjusted to maintain saturations ≥ 90%). The decisions to intubate a patient, escalate HFNC settings, or switch NIV interfaces were at the discretion of an attending physician guided by examination and, at times, available radiographic and laboratory data. Patients receiving HFNC flow rates > 4 L/min were monitored in the PICU. After a determination of clinical stability was made during which a patient was maintained on ≤ 6 L/min, they were considered for transfer to the general pediatric floor from the PICU. If a patient's flow rate was weaned to 4 L/min, they were subsequently transitioned to a regular nasal cannula.

## Results

### General Sample Characteristics

**Figure 3:**
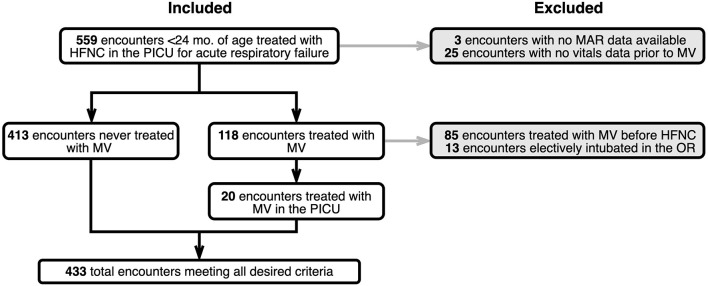
CONSORT diagram depicting study criteria and sampling.

A total of 433 children were included in the study. Figure 3 shows a CONSORT diagram depicting study sampling and criteria. Descriptive characteristics for the study population and for cohorts defined by the presence of one or more HFNC flow rate escalations are depicted in Table 1. Of note, children with escalations were more frequently Black (38.8% vs. 23.5, *P* = 0.046), had a lower mean initial HFNC flow rate (0.8 ± 0.6 L/kg/min vs. 1.1 ± 0.5 L/kg/min, *P* < 0.001), and experienced a longer median hospital length of stay [4.3 (IQR:2.9, 7.5) vs. 2.5 (IQR:1.7, 3.5) days, *P* < 0.001]. Duration of HFNC was also longer in patients with flow rate escalation [42.0 (IQR: 22.4–70.2) vs. 19.5 (IQR: 10.7–36.2) h, *P* < 0.001]. Children treated with at least a 7 day course of antibiotics all had at least one instance of flow rate escalation (8.1% of escalated patients vs. 0% of non-escalated patients, *P* < 0.001). The maximum flow rate given to patients with at least one instance of escalation was 1.3 L/kg/min (IQR: 1.0–1.8 L/kg/min) vs. 1.0 L/kg/min (IQR: 0.7–1.4 L/kg/min) for patients who were never escalated (*P* < 0.001).

### Effect of Time Window on Model Performance

**Figure 4:**
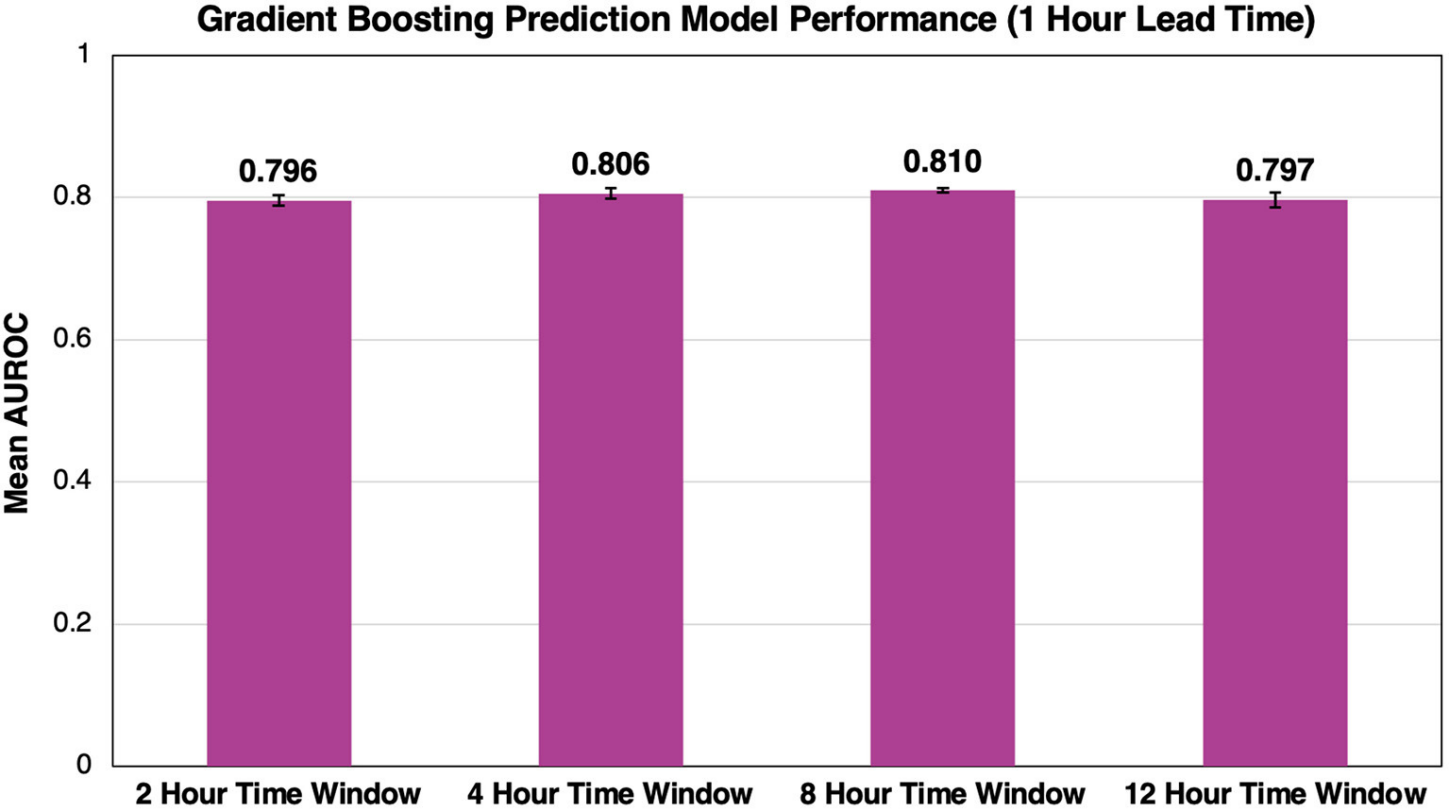
Effect of time window size on AUROC performance. As we increase the time window size, we see minimal impact on the overall success of the model. Error bars represent 95% confidence intervals between our five cross-validation folds.

Figure 4 shows the average AUROC for our best-performing gradient boosting model at each time window for five cross-validation folds. Given the evaluation of time window size on model performance, lead times were constant at 1 h before flow rate escalation. Overall, time window size had little impact on model performance as windows of 2, 4, 8, and 12 h had AUROCs of 0.796 ± 0.007, 0.806 ± 0.007, 0.810 ± 0.003, and 0.797 ± 0.011, respectively. A time window of 8 h was selected because it outperformed all other windows for each lead time evaluated.

### Effect of Lead Time on Model Performance

**Figure 5:**
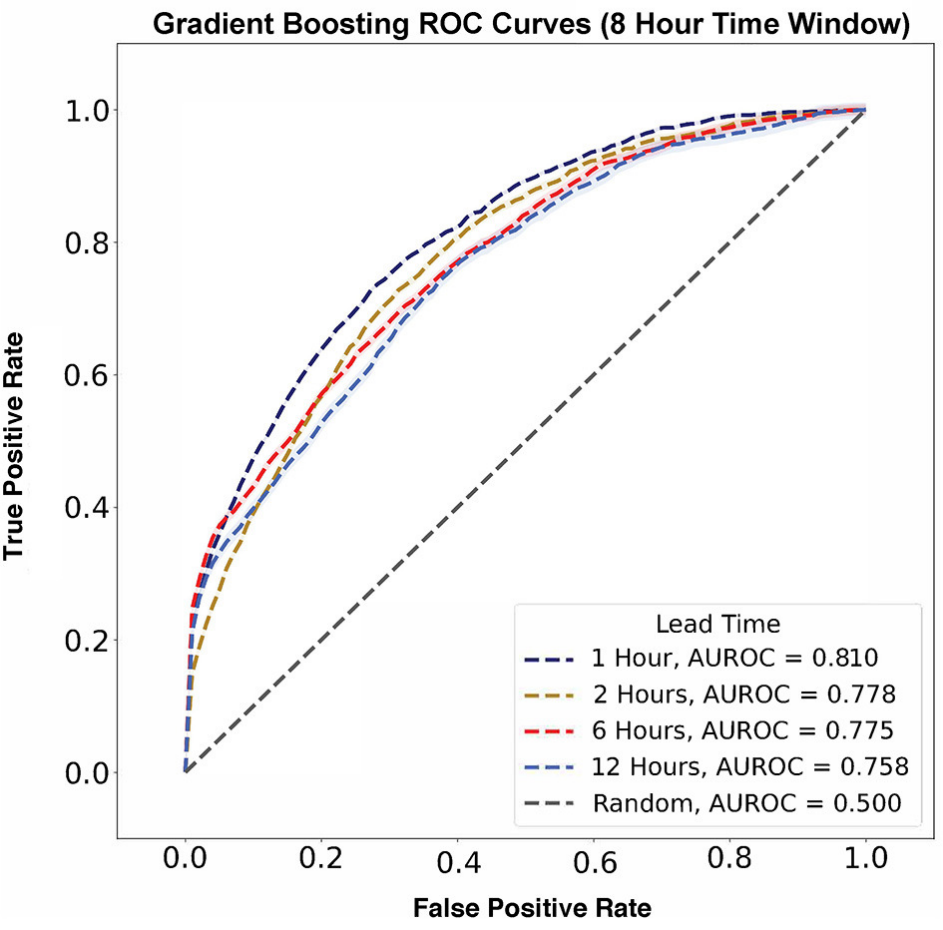
Flow rate escalation ROCs with shaded 95% confidence interval bounds for gradient boosting algorithms with a time window of 8 h. The model has its largest AUROC value of 0.810 at a lead time of 1 h and decreases consistently as lead time increases.

Using the 8 h time window, model performance was evaluated across all lead times. The best performing model had a lead time of 1 h and an AUROC of 0.810 ± 0.003 (Figure 5). As lead time increased, AUROC decreased from 0.778 ± 0.007 to 0.775 ± 0.009 and 0.758 ± 0.012 for 2, 6, and 12 h, respectively, before flow rate escalation. Thus, a lead time of 1 h outperformed all other lead times.

**Figure 6:**
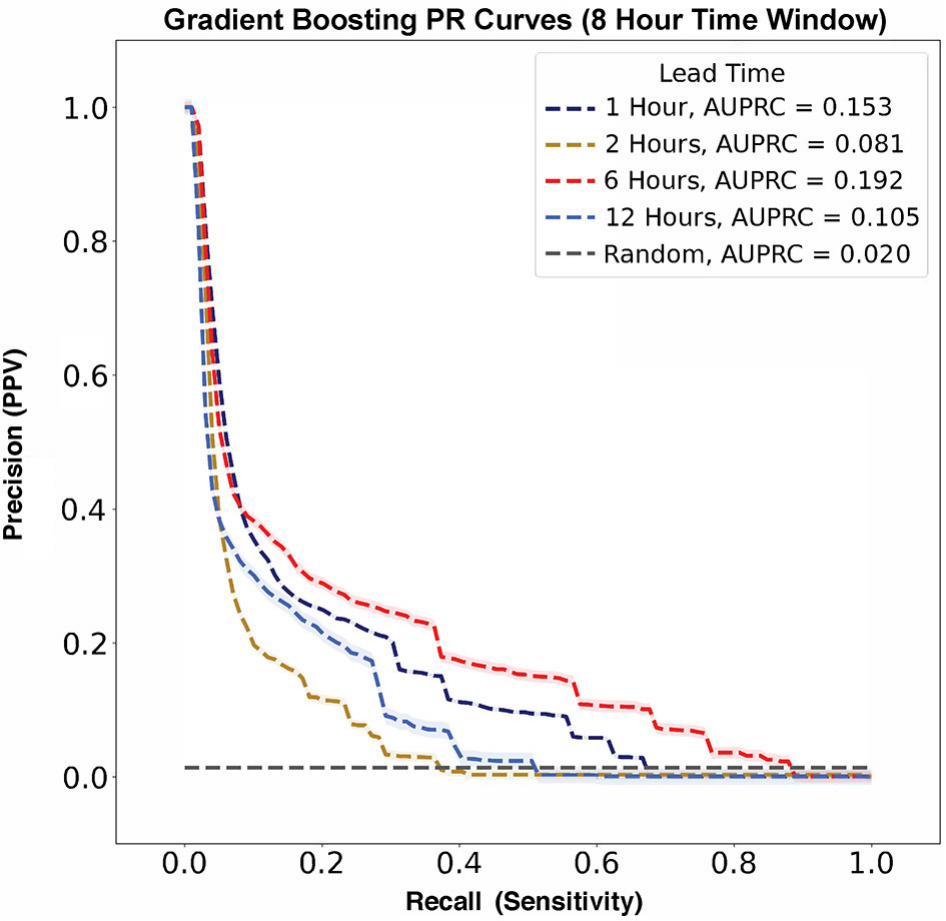
Flow rate escalation precision-recall curves with shaded 95% confidence interval bounds for gradient boosting algorithms with a time window of 8 h. The model has its largest AUPRC value of 0.192 at a lead time of 6 h. There is not a clear trend between AUPRC and lead time. For reference, the prevalence of flow rate escalations in our model was 0.020, as represented by the gray no-skill classifier line.

In contrast, AUPRC data did not show any trend across varying lead times (Figure 6). The largest AUPRC, 0.192 ± 0.009, was noted for a lead time of 6 h. For lead times of 1, 12, and 2 h, the AUPRC values were 0.153 ± 0.003, 0.105 ± 0.012, and 0.081 ± 0.007, respectively.

### Comparison of Model Performance to ROX and ROX-HR Indices

**Figure 7:**
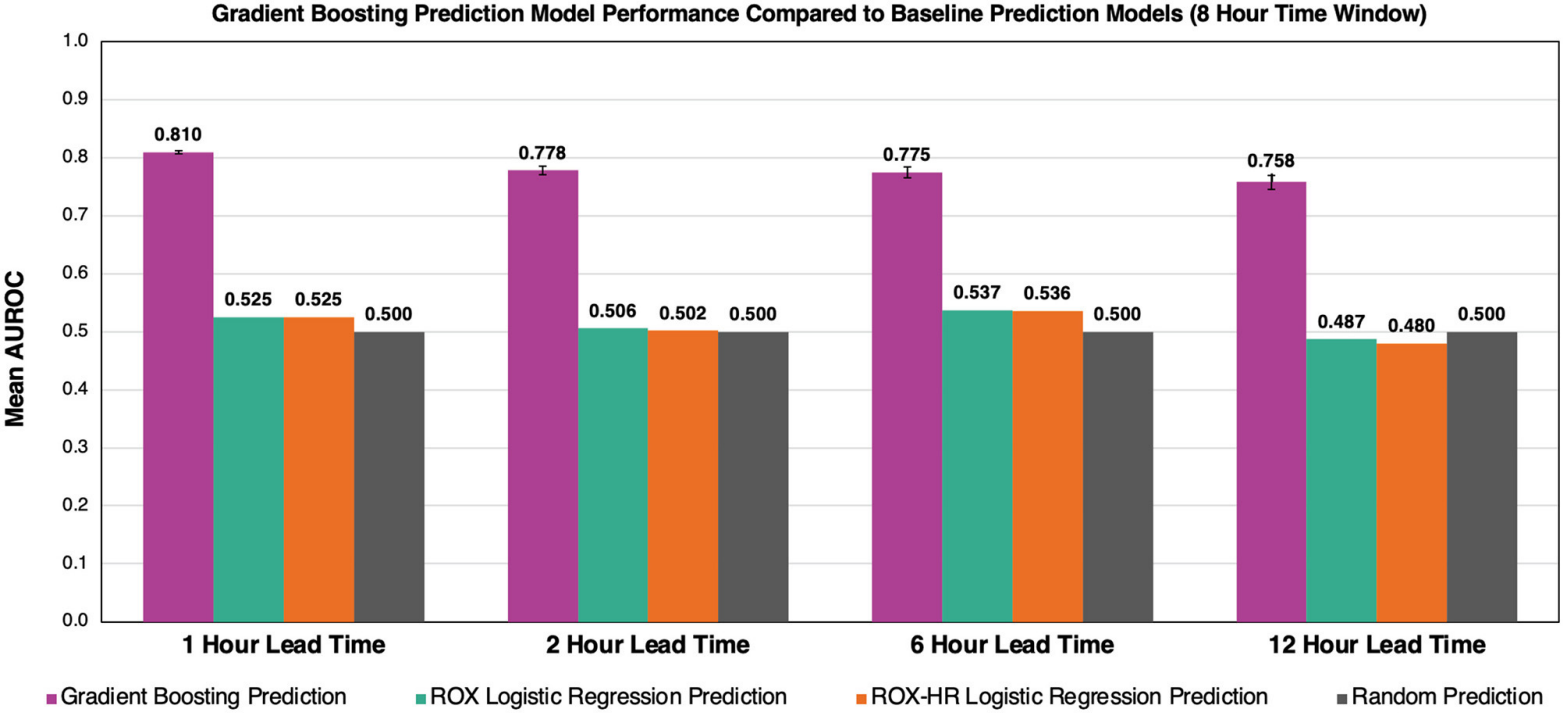
Comparison of gradient boosting model to ROX and ROX-HR with respect to AUROC performance. At a time window of 8 h, our model outperforms the ROX and ROX-HR logistic regression baselines at lead times of 1, 2, 6, and 12 h. Error bars represent 95% confidence intervals between our five cross-validation folds. Error bars for ROX and ROX-HR predictions are not visible and are zero up to three decimals.

The gradient boosting model outperformed the ROX and ROX-HR logistic regression models (Figure 7). Using a time window of 8 h, the gradient boosting model had AUROC values of 0.810 ± 0.003, 0.778 ± 0.007, 0.775 ± 0.009, and 0.758 ± 0.012 with lead times of 1, 2, 6, and 12 h, respectively. In comparison, the ROX yielded AUROC values of 0.525 ± 0.000, 0.506 ± 0.000, 0.537 ± 0.000, and 0.487 ± 0.000. The ROX-HR yielded AUROC values of 0.525 ± 0.000, 0.502 ± 0.000, 0.536 ± 0.000, and 0.480 ± 0.000.

### Risk Score Trajectories Throughout Patient Stay

**Figure 8:**
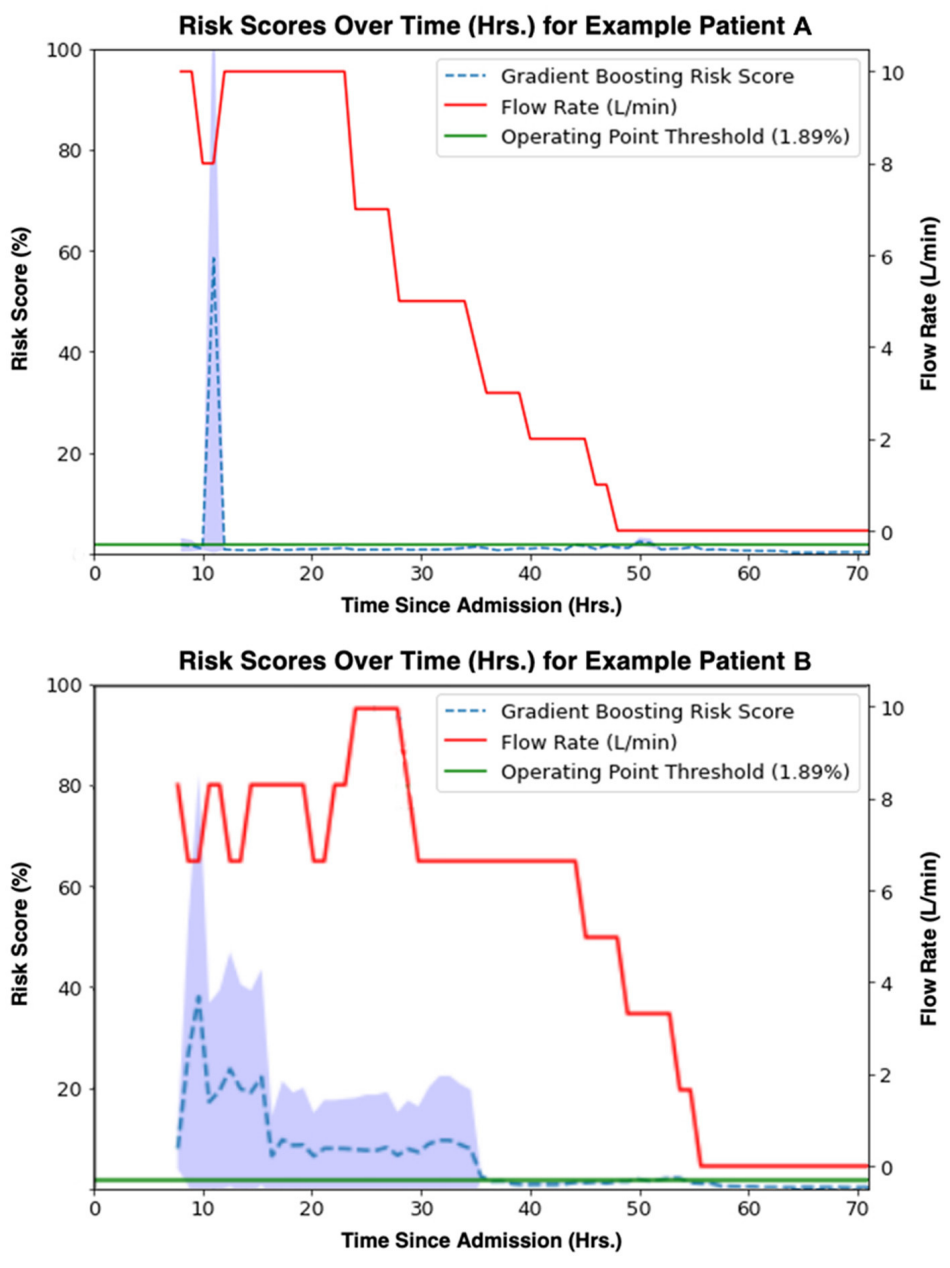
Gradient boosting model (time window = 8 h, lead time = 1 h) risk scores [%] (blue) aligned with true flow rate [L/min] (red) for two example patients' observation periods. A threshold (1.89%) at the model operating point is shown in green.

The study model's prediction probabilities (as percentages) along with a patient's set flow rate for each hour of their hospital stay were plotted to visualize an individual patient's predicted escalation status over time. Visualizations for two example patients, Example Patient A and Example Patient B, are available in Figure 8. An increase in risk score closely preceding an escalation in flow rate qualitatively reflects the model's performance. In Figure 8, Example Patient A underwent an attempted wean 10 h after their initiation on HFNC, and our model's risk score appropriately rose, predicting their re-escalation of flow rate. On the other hand, Example Patient B was managed at too low of a flow rate with a persistently elevated risk score before an appropriate flow rate was set, and then the patient was safely weaned.

### Feature Importance Rankings

Shapley values for each feature at five cross-validation folds were calculated to identify variables that contributed heavily to the model outcome. Highly ranked features, as shown in both Tables 2, 3, indicate those that the model deems important to make a flow rate escalation prediction. Table 2 displays the top twelve features within our model for each lead time. A majority of these top features derived from HFNC settings, vital sign data, the ROX index, the number of times F_i_O_2_ was adjusted, and the newly-established over-saturation label. Table 3 compares the synthetic features (ROX index, ROX-HR index, number of times F_i_O_2_ was modified, and the over-saturation label) at each lead time.

**Table 2:** Top twelve feature importance rankings based on Shapley values.

**Shapley Feature Importance Rank**	**1 H Lead Time**	**2 H Lead Time**	**6 H Lead Time**	**12 H Lead Time**
1	Number of Times Respiratory Rate Recorded	Number of Times Respiratory Rate Recorded	Number of Times Pulse Recorded	Number of Times Pulse Recorded
2	Mean ROX-HR Index	Minimum F_i_O_2_	S_p_O_2_ Standard Deviation	S_p_O_2_ Standard Deviation
3	Last Recorded F_i_O_2_	Number of Times Pulse Recorded	Minimum F_i_O_2_	Minimum F_i_O_2_
4	Number of Times Pulse Recorded	S_p_O_2_ Standard Deviation	Oversaturation Label: F_i_O_2_ ≥ 60 & S_p_O_2_ ≥ 97	Number of Times F_i_O_2_Changes
5	Mean ROX Index	Oversaturation Label: F_i_O_2_ ≥ 60 & S_p_O_2_ ≥ 97	Mean S_p_O_2_	Mean S_p_O_2_
6	S_p_O_2_ Standard Deviation	Number of Times F_i_O_2_ Changes	Number of Times F_i_O_2_ Changes	Number of Times Respiratory Rate Recorded
7	Number of Times F_i_O_2_ Changes	Mean ROX Index	F_i_O_2_ Standard Deviation	Mean ROX Index
8	Number of Times F_i_O_2_ Recorded	Number of Times S_p_O_2_ Recorded	Last Recorded F_i_O_2_	Oversaturation Label: F_i_O_2_ ≥ 60 & S_p_O_2_ ≥ 97
9	F_i_O_2_ Standard Deviation	Mean F_i_O_2_	Mean ROX-HR Index	Mean SBP
10	Mean Respiratory Rate	Change in S_p_O_2_	Number of Times Respiratory Rate Recorded	Mean Respiratory Rate
11	Oversaturation Label: F_i_O_2_ ≥ 60 & S_p_O_2_ ≥ 97	F_i_O_2_ Standard Deviation	Mean ROX Index	Median SBP
12	Change in S_p_O_2_	Last Recorded F_i_O_2_	Minimum DBP	Respiratory Rate Standard Deviation

*At a time window of 8 h, we ran the model on five parallel validation folds for each lead time and took the average Shapley value of each feature as a ranking. Synthetic features are indicated with a colored box*.

**Table 3:** Feature importances of our four synthetic features.

**Feature**	**1 H Lead Time**	**2 H Lead time**	**6 H Lead Time**	**12 H Lead Time**
**Number of F**_**i**_**O**_**2**_ **Changes**				
Rank (Range)	7 (2–7)	6 (4–6)	6 (3–6)	4 (4–8)
Shapley Value (CI)	0.136 ± 0.027	0.150 ± 0.018	0.116 ± 0.015	0.093 ± 0.009
**Oversaturation Label: F_i_O_2_ ≥ 60, S_p_O_2_ ≥ 97**				
Rank (Range)	11 (9–14)	5 (4–8)	4 (2–6)	8 (5–41)
Shapley Value (CI)	0.062 ± 0.014	0.155 ± 0.067	0.137 ± 0.019	0.054 ± 0.028
**Mean ROX Index**				
Rank (Range)	5 (1–46)	7 (3–46)	11 (6–20)	7 (3–83)
Shapley Value (CI)	0.156 ± 0.135	0.114 ± 0.118	0.065 ± 0.043	0.064 ± 0.051
**Mean ROX-HR Index**				
Rank (Range)	2 (3-9)	14 (9–28)	9 (4–14)	19 (14–27)
Shapley Value (CI)	0.181 ± 0.087	0.055 ± 0.028	0.085 ± 0.052	0.030 ± 0.013

*The rank is based on the average Shapley value of 5 cross-validation folds, while the range spans the synthetic feature's importance between all five-folds. The average Shapley value and 95% confidence interval for each of the four synthetic features are listed to describe the spread between folds*.

## Discussion

### Major Findings

In this study, we demonstrate the ability of tree-based machine learning models, namely gradient boosting, to predict subsequent escalation of HFNC flow rate with a specified lead within the PICU patient population using time-series vital sign data and electronic health records. Our work represents the first attempt to develop models to predict HFNC flow rate escalation instead of subsequent escalation to MV. Additionally, we present the first characterization of prediction metrics previously validated for MV escalation adapted for flow rate escalation and demonstrate that our methods outperform these existing metrics. The determination of sufficient or appropriate HFNC settings for pediatric patients with acute respiratory failure remains unknown (30, 31). Clinicians in the PICU must balance providing adequate and timely NIV support to a patient's dynamic pathophysiology while avoiding invasive and potentially harmful interventions. These methods may offer the potential to optimize, individualize, and even automize NIV parameters for critically ill children with acute respiratory failure.

### Comparisons With ROX and Machine Learning Methods

Previous definitions used for HFNC non-response have hinged on either identifying patients with an escalation to MV or transition to non-invasive positive pressure ventilation (NIPPV) (11, 18, 19). The ROX index was initially established by Roca et al. as a metric for MV prediction in a prospective cohort of adults with pneumonia and has been widely explored in various conditions, including COVID-19 pneumonia and acute respiratory failure in immunocompromised adults (32, 33). In their clinical validation study, Roca et al. evaluated ROX index prediction thresholds at 2, 6, and 12 h after HFNC initiation in a validation cohort of 191 adult patients in which 36% required endotracheal intubation, finding AUROCs of 0.68, 0.70, and 0.76, respectively (18). We made inferences using similar time scales with time windows of 2, 4, 8, and 12 (AUROCs = 0.80, 0.81, 0.81, and 0.80, respectively) to predict flow rate escalation (34). However, unlike our model, the ROX index does not explicitly consider a lead time to escalation but instead predicts any future instance of mechanical ventilation following a prediction-score calculation. While time-to-intubation is not directly discussed in their clinical validation study, in their initial study, Roca et al. states that the median time-to-intubation for their cohort was 1 day, aligning with their highest AUROC prediction score calculated at 24 h from HFNC initiation (18). We chose to consider lead time when defining flow rate escalation instances as the physiologic signals, such as increased work of breathing, are likely to be temporally linked to escalation. Indeed, our AUROCs decrease as lead time increases, demonstrating that earlier predictions are more difficult to identify than predictions closer to the time of escalation and supporting the construct validity of our model. Furthermore, incorporating lead time into our model offers greater interpretability for when an escalation is likely to occur, allowing a provider to determine the urgency and degree of intervention required.

In order to explore and compare the ROX index for flow rate escalation prediction, we felt it was unreasonable to utilize existing ROX cutoff thresholds for flow rate escalation prediction. Predicting flow rate escalation at a given lead time in a pediatric population fundamentally differs from the experiments Roca et al. performed. Instead, we re-fit a logistic regression using only the ROX index to predict flow rate escalation. This ROX-based model had the best AUROC of 0.54 considering 8 h time windows of HFNC data at a lead time of 6 h. Goh et al. (19) posited that the ROX-HR index had promising utility for predicting MV in post-extubation patients on HFNC, achieving an AUROC of 0.72 and 0.74 at 10 h for the ROX and ROX-HR indices, respectively. We adapted the ROX-HR index in a similar method as the ROX index and found that the AUROC for flow rate escalation prediction at a specified lead time was lower than our machine learning model but equal to the predictions based on the ROX index alone, with a best AUROC of 0.54 when considering 8 h of HFNC data at lead time of 6 h. These results demonstrate that the ROX and ROX-HR indexes have good performance in predicting MV but are less capable of independently discriminating between patients who require flow rate escalations at a specified lead time.

Lundberg et al. similarly found that gradient boosting methods had superior performance in predicting intraoperative hypoxemia to other machine learning methods, such as SVM and lasso regression, and employed Shapely feature values when improving their model's interpretability for real-time clinical decision making (35). Previous attempts have explored the prediction of invasive MV weaning outcomes in adult patients using deep learning approaches (36, 37). Neural networks have also been investigated in the pediatric patient population to predict acute severe asthma exacerbations (38). These works differ in that some approaches use only time-series data and others utilize static patient-averaged variables. Comparatively, our modeling approach benefits from using both time-series and static variables and achieves good predictive performance; however, these other works provide an outline for future modeling directions with high-resolution vitals data and neural network-based approaches.

### Secondary Findings

Descriptive analysis of our patient data found significant differences in race composition between escalated and non-escalated encounters. One possible explanation for these findings is that there are known racial disparities in viral respiratory hospitalizations in children, and these disparities could also relate to a higher likelihood for providers to escalate flow rates in Black children than White children (39). Although our model had access to racial demographic data, these features were not ranked highly within our Shapely feature importances, suggesting that these racial disparities do not heavily inform our model's prediction of flow rate escalations at a given lead time. Additionally, we found the starting flow rate given to patients who never escalate is greater than the flow rate given to those who escalate with a mean difference between these groups of 1.6 L/min (0.3 L/kg/min). Patients who never escalate also have a shorter duration of high flow use. This may be related to the day of illness at presentation. Symptoms of acute respiratory failure typically peak during the third to fifth day of illness before improving. Patients presenting prior to peak symptoms might require less support initially but must be escalated initially as symptoms worsen. Patients presenting later at or after peak symptoms would require higher initial settings, but then de-escalation as symptoms improve.

When evaluating our model's feature importance through Shapley additive explanations, we found that features representing physiologic vitals data and F_i_O_2_ parameters were considered important. These findings are consistent with previous reports demonstrating the importance of persistent vital sign abnormalities and other comorbidities (i.e., concurrent bacterial pneumonia, persistent hypercarbia, and history of prematurity) as likely indicators of NIV non-response (10, 13, 40–42). Despite their poor performance predicting flow rate escalation in independent models, the ROX and ROX-HR features within our prediction model were highly valuable. This suggests the ROX and ROX-HR scores have value as indices for flow rate escalation when features that infer provider perception of disease severity and attention to weaning are incorporated (i.e., F_i_O_2_ changes and the oversaturation label).

### Limitations and Future Studies

Our data represent a retrospective assessment with potential bias from errors in documentation within the EHR. As this was a single-center experience, observations may be due to individual provider or regional/institutional variation in acute respiratory failure and HFNC management. Additionally, it is impossible for us to know what specific factors affected provider decision making with regards to escalating HFNC flow rate for a given patient. Provider knowledge of vital signs and physiologic monitoring were unknown and may have influenced such outcomes. Study data were not recorded specifically for the purpose of applying machine learning methods, which led to potential error from inclusion of data imputation and filling. This need could be avoided by setting prospective protocols regarding minimum synchronized vitals and flow rate parameter recording intervals for future investigation. Synthesized variables, such as the number of vitals recorded within a time window or F_i_O_2_ settings, are inherently dependent on clinician perception of disease severity, not measured in this study. These feature's inclusion is consistent with previous prediction tools, like the ROX index, but leaves room for bias from provider or regional variation. These factors may limit the generalizability of this model to dissimilar institutions or centers with variation in HFNC practices such as use of greater standard flow rates or application of the device in step-down ICUs or general pediatric wards. Future studies will be needed with prospective analysis and external validation before any conclusions can be drawn about future bedside applicability. Lastly, while our model potentially could be used to predict whether a patient who has been weaned is likely to remain stable or require re-escalation, we note that this work is not directed toward prediction of weaning success. Our focus was on predicting HFNC escalation to best compare performance to the ROX score. Prediction of weaning success is a clinically important task and a worthwhile objective for future models.

### Conclusion

In conclusion, our retrospective study demonstrates that machine learning models can discriminate subsequent HFNC flow rate escalation among children under 2 years of age admitted for acute respiratory failure. As a majority of data included in the study model are readily accessible from electronic health record data, these models could be replicated at other institutions and employed prospectively to assist with provider assessment of clinical trajectory, add to informed decision making, and suggest suitable HFNC parameters. Ultimately, use of this and similar models may narrow the gap between identifying the need for increased support and applying a sufficient degree of it. These efforts may then impact hospital length of stay and other clinical outcomes in future investigation.

## Data Availability Statement

The datasets presented in this article are not readily available because HIPAA restrictions prevent its distribution. Requests to access the datasets should be directed to jim@jhmi.edu.

## Ethics Statement

The studies involving human participants were reviewed and approved by Johns Hopkins Medical Institutional Review Board. Written informed consent from the participant's legal guardian/next of kin was not required to participate in this study in accordance with the national legislation and the institutional requirements.

## Author Contributions

Our team of co-lead authors performed experimental design, execution, and analysis, for which JK was the team leader. Clinical guidance was provided by JB, JF, and AS. Data modeling guidance was given by KG, IG, RW, and JG. All authors contributed to the article and approved the submitted version.

## Conflict of Interest

The authors declare that the research was conducted in the absence of any commercial or financial relationships that could be construed as a potential conflict of interest.

## Publisher's Note

All claims expressed in this article are solely those of the authors and do not necessarily represent those of their affiliated organizations, or those of the publisher, the editors and the reviewers. Any product that may be evaluated in this article, or claim that may be made by its manufacturer, is not guaranteed or endorsed by the publisher.

## Supplementary Material

Supplementary Figure 1 Class imbalance in cohort data. Because the ratio remains stable across all time windows, we determined that our dataset was balanced enough to not have to use undersampling, oversampling, or generate synthetic samples.

Supplementary Figure 2 Receiver operating characteristic curves comparing prediction models to baseline models for each lead time at a time window of 2 h. AUROCs for the logistic regression, random forest, and gradient boosting models are compared to baseline ROX logistic regression, ROX-HR logistic regression, and random prediction models.

Supplementary Figure 3 Receiver operating characteristic curves comparing prediction models to baseline models for each lead time at a time window of 4 h. AUROCs for the logistic regression, random forest, and gradient boosting models are compared to baseline ROX logistic regression, ROX-HR logistic regression, and random prediction models.

Supplementary Figure 4 Receiver operating characteristic curves comparing prediction models to baseline models for each lead time at a time window of 8 h. AUROCs for the logistic regression, random forest, and gradient boosting models are compared to baseline ROX logistic regression, ROX-HR logistic regression, and random prediction models.

Supplementary Figure 5 Receiver operating characteristic curves comparing prediction models to baseline models for each lead time at a time window of 12 h. AUROCs for the logistic regression, random forest, and gradient boosting models are compared to baseline ROX logistic regression, ROX-HR logistic regression, and random prediction models.

Supplementary Figure 6 Shapley summary plot for our four synthetic features. Shapley additive explanations calculate the feature relevance for each individual prediction independently and depict it as a point in the figure. For each feature, this plot shows how a higher or lower value of that feature, represented by color, influences whether the model predicts a flow rate escalation (positive Shapley value) or not (negative Shapley value). The magnitude of the Shapley value indicates how strongly the model agrees with this prediction.

Supplementary Table 1 Average starting values of oxygen flow rate and fraction of inspired oxygen for each level of respiratory support.

Supplementary Table 2 All of the features used in our model.

Supplementary Table 3 Performance metrics comparing gradient boosting, ROX, and ROX-HR model performances to a random classifier at a time window of 8 h. The metrics include sensitivity, specificity, mean AUROC, mean AUPRC, positive predictive value (PPV), and negative predictive value (NPV). The PPV for ROX and ROX-HR logistic regression models could not be obtained, because these models predicted no true positive and false positive values.
