# Supplementary figures and images for: Predicting Flow Rate Escalation for Pediatric Patients on High Flow Nasal Cannula Using Machine Learning
Source: Front Pediatr. 2021 Nov 8;9:734753. doi: 10.3389/fped.2021.734753 (PMC8606666; doi:10.3389/fped.2021.734753)

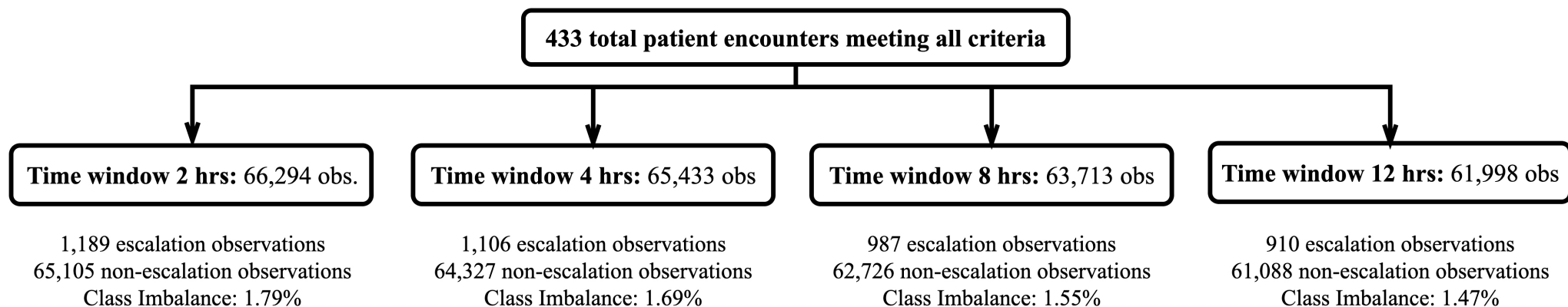

Supplement: Supplementary Figure 1 — Class imbalance in cohort data. Because the ratio remains stable across all time windows, we determined that our dataset was balanced enough to not have to use undersampling, oversampling, or generate synthetic samples. [file Image_1.PDF]
