# Supplementary material for: Predicting Flow Rate Escalation for Pediatric Patients on High Flow Nasal Cannula Using Machine Learning
Source: Front Pediatr. 2021 Nov 8;9:734753. doi: 10.3389/fped.2021.734753 (PMC8606666; doi:10.3389/fped.2021.734753)
Supplement: Supplementary Figure 2 — Receiver operating characteristic curves comparing prediction models to baseline models for each lead time at a time window of 2 h. AUROCs for the logistic regression, random forest, and gradient boosting models are compared to baseline ROX logistic regression, ROX-HR logistic regression, and random prediction models. [file Image_2.PDF]

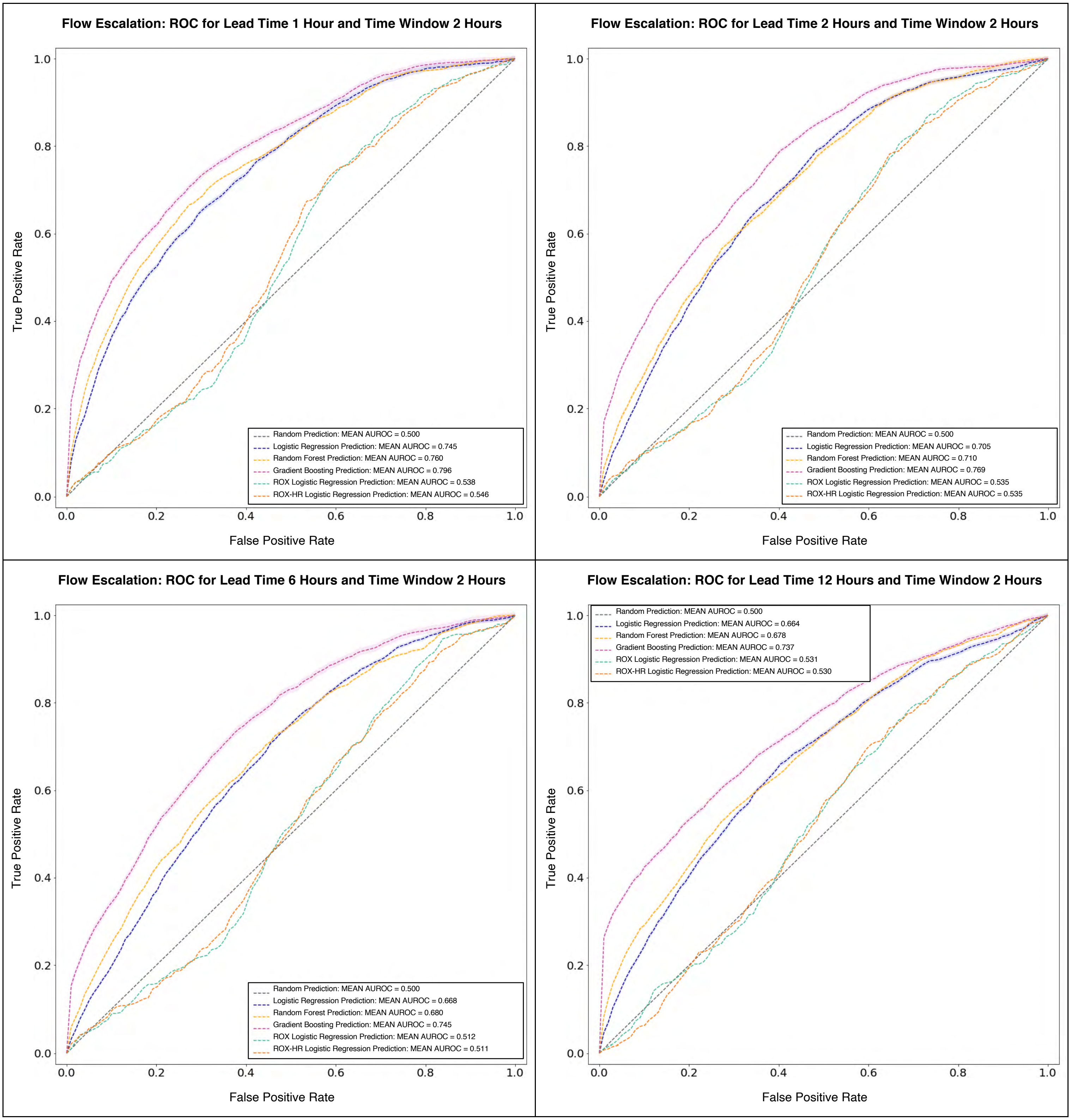

Flow Escalation: ROC for Lead Time 1 Hour and Time Window 2 Hours
True Positive Rate
False Positive Rate
Random Prediction: MEAN AUROC = 0.500
Logistic Regression Prediction: MEAN AUROC = 0.745
Random Forest Prediction: MEAN AUROC = 0.760
Gradient Boosting Prediction: MEAN AUROC = 0.796
ROX Logistic Regression Prediction: MEAN AUROC = 0.538
ROX-HR Logistic Regression Prediction: MEAN AUROC = 0.546
Flow Escalation: ROC for Lead Time 2 Hours and Time Window 2 Hours
True Positive Rate
False Positive Rate
Random Prediction: MEAN AUROC = 0.500
Logistic Regression Prediction: MEAN AUROC = 0.705
Random Forest Prediction: MEAN AUROC = 0.710
Gradient Boosting Prediction: MEAN AUROC = 0.769
ROX Logistic Regression Prediction: MEAN AUROC = 0.535
ROX-HR Logistic Regression Prediction: MEAN AUROC = 0.535
Flow Escalation: ROC for Lead Time 6 Hours and Time Window 2 Hours
True Positive Rate
False Positive Rate
Random Prediction: MEAN AUROC = 0.500
Logistic Regression Prediction: MEAN AUROC = 0.668
Random Forest Prediction: MEAN AUROC = 0.680
Gradient Boosting Prediction: MEAN AUROC = 0.745
ROX Logistic Regression Prediction: MEAN AUROC = 0.512
ROX-HR Logistic Regression Prediction: MEAN AUROC = 0.511
Flow Escalation: ROC for Lead Time 12 Hours and Time Window 2 Hours
True Positive Rate
False Positive Rate
Random Prediction: MEAN AUROC = 0.500
Logistic Regression Prediction: MEAN AUROC = 0.664
Random Forest Prediction: MEAN AUROC = 0.678
Gradient Boosting Prediction: MEAN AUROC = 0.737
ROX Logistic Regression Prediction: MEAN AUROC = 0.531
ROX-HR Logistic Regression Prediction: MEAN AUROC = 0.530
