# Supplementary material for: Predicting Flow Rate Escalation for Pediatric Patients on High Flow Nasal Cannula Using Machine Learning
Source: Front Pediatr. 2021 Nov 8;9:734753. doi: 10.3389/fped.2021.734753 (PMC8606666; doi:10.3389/fped.2021.734753)
Supplement: Supplementary Figure 3 — Receiver operating characteristic curves comparing prediction models to baseline models for each lead time at a time window of 4 h. AUROCs for the logistic regression, random forest, and gradient boosting models are compared to baseline ROX logistic regression, ROX-HR logistic regression, and random prediction models. [file Image_3.PDF]

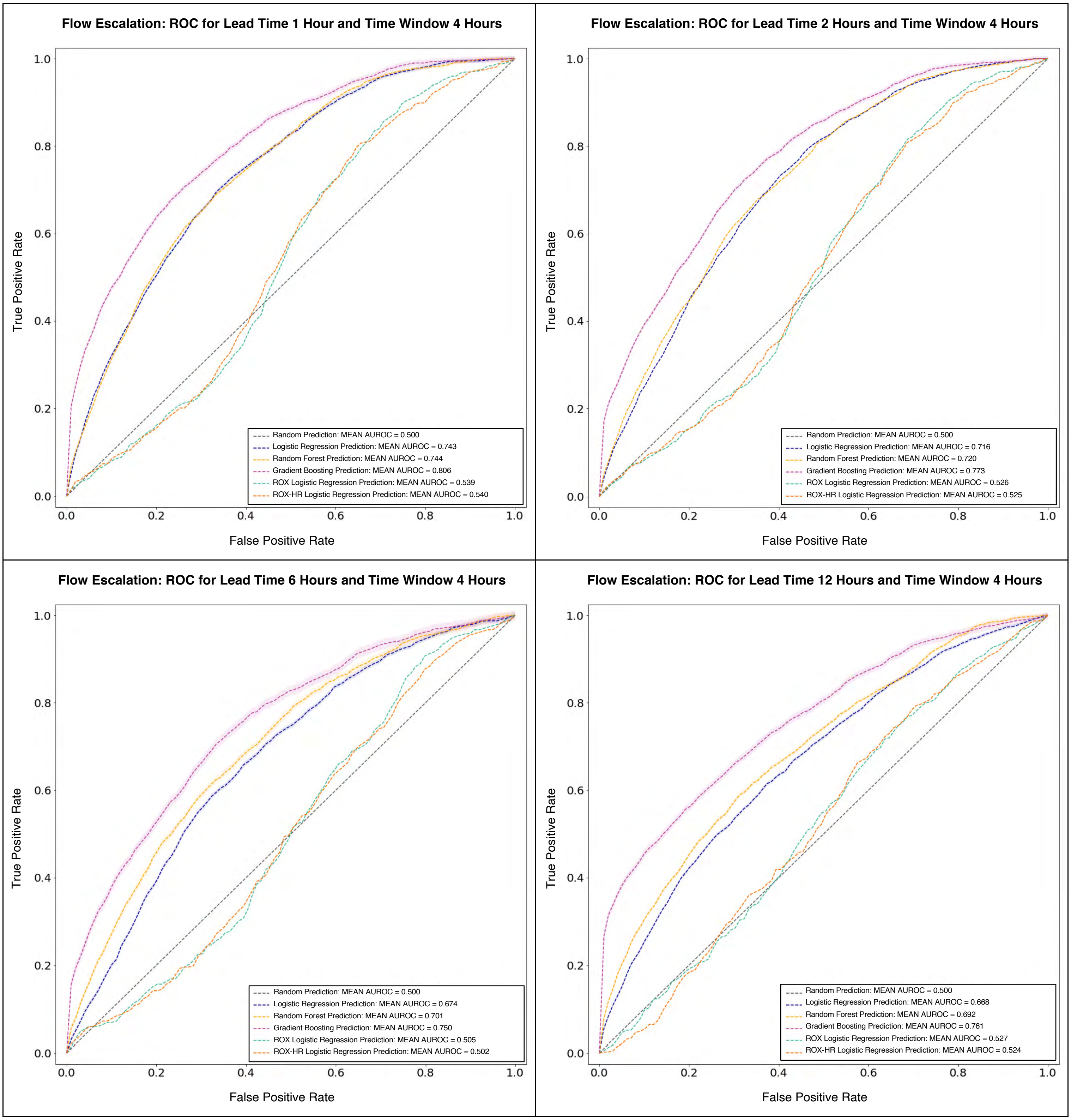
Flow Escalation: ROC for Lead Time 1 Hour and Time Window 4 Hours
True Positive Rate
False Positive Rate
0.0
0.2
0.4
0.6
0.8
1.0
Random Prediction: MEAN AUROC = 0.500
Logistic Regression Prediction: MEAN AUROC = 0.743
Random Forest Prediction: MEAN AUROC = 0.744
Gradient Boosting Prediction: MEAN AUROC = 0.806
ROX Logistic Regression Prediction: MEAN AUROC = 0.539
ROX-HR Logistic Regression Prediction: MEAN AUROC = 0.540
Flow Escalation: ROC for Lead Time 2 Hours and Time Window 4 Hours
True Positive Rate
False Positive Rate
Random Prediction: MEAN AUROC = 0.500
Logistic Regression Prediction: MEAN AUROC = 0.716
Random Forest Prediction: MEAN AUROC = 0.720
Gradient Boosting Prediction: MEAN AUROC = 0.773
ROX Logistic Regression Prediction: MEAN AUROC = 0.526
ROX-HR Logistic Regression Prediction: MEAN AUROC = 0.525
Flow Escalation: ROC for Lead Time 6 Hours and Time Window 4 Hours
True Positive Rate
False Positive Rate
Random Prediction: MEAN AUROC = 0.500
Logistic Regression Prediction: MEAN AUROC = 0.674
Random Forest Prediction: MEAN AUROC = 0.701
Gradient Boosting Prediction: MEAN AUROC = 0.750
ROX Logistic Regression Prediction: MEAN AUROC = 0.505
ROX-HR Logistic Regression Prediction: MEAN AUROC = 0.502
Flow Escalation: ROC for Lead Time 12 Hours and Time Window 4 Hours
True Positive Rate
False Positive Rate
Random Prediction: MEAN AUROC = 0.500
Logistic Regression Prediction: MEAN AUROC = 0.668
Random Forest Prediction: MEAN AUROC = 0.692
Gradient Boosting Prediction: MEAN AUROC = 0.761
ROX Logistic Regression Prediction: MEAN AUROC = 0.527
ROX-HR Logistic Regression Prediction: MEAN AUROC = 0.524
