# Supplementary material for: Predicting Flow Rate Escalation for Pediatric Patients on High Flow Nasal Cannula Using Machine Learning
Source: Front Pediatr. 2021 Nov 8;9:734753. doi: 10.3389/fped.2021.734753 (PMC8606666; doi:10.3389/fped.2021.734753)
Supplement: Supplementary Figure 4 — Receiver operating characteristic curves comparing prediction models to baseline models for each lead time at a time window of 8 h. AUROCs for the logistic regression, random forest, and gradient boosting models are compared to baseline ROX logistic regression, ROX-HR logistic regression, and random prediction models. [file Image_4.PDF]

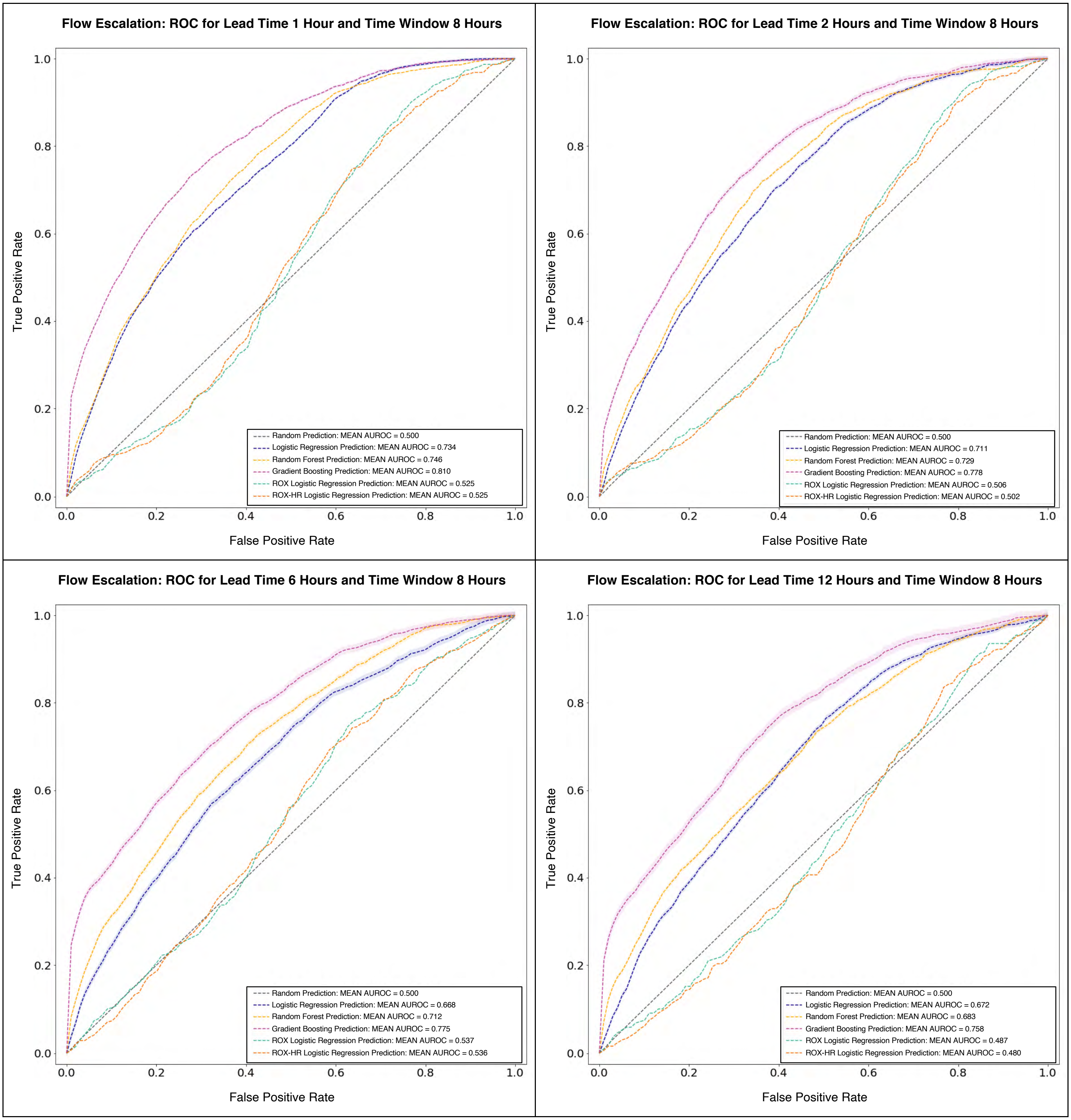

Flow Escalation: ROC for Lead Time 1 Hour and Time Window 8 Hours
True Positive Rate
False Positive Rate
Random Prediction: MEAN AUROC = 0.500
Logistic Regression Prediction: MEAN AUROC = 0.734
Random Forest Prediction: MEAN AUROC = 0.746
Gradient Boosting Prediction: MEAN AUROC = 0.810
ROX Logistic Regression Prediction: MEAN AUROC = 0.525
ROX-HR Logistic Regression Prediction: MEAN AUROC = 0.525
Flow Escalation: ROC for Lead Time 2 Hours and Time Window 8 Hours
True Positive Rate
False Positive Rate
Random Prediction: MEAN AUROC = 0.500
Logistic Regression Prediction: MEAN AUROC = 0.711
Random Forest Prediction: MEAN AUROC = 0.729
Gradient Boosting Prediction: MEAN AUROC = 0.778
ROX Logistic Regression Prediction: MEAN AUROC = 0.506
ROX-HR Logistic Regression Prediction: MEAN AUROC = 0.502
Flow Escalation: ROC for Lead Time 6 Hours and Time Window 8 Hours
True Positive Rate
False Positive Rate
Random Prediction: MEAN AUROC = 0.500
Logistic Regression Prediction: MEAN AUROC = 0.668
Random Forest Prediction: MEAN AUROC = 0.712
Gradient Boosting Prediction: MEAN AUROC = 0.775
ROX Logistic Regression Prediction: MEAN AUROC = 0.537
ROX-HR Logistic Regression Prediction: MEAN AUROC = 0.536
Flow Escalation: ROC for Lead Time 12 Hours and Time Window 8 Hours
True Positive Rate
False Positive Rate
Random Prediction: MEAN AUROC = 0.500
Logistic Regression Prediction: MEAN AUROC = 0.672
Random Forest Prediction: MEAN AUROC = 0.683
Gradient Boosting Prediction: MEAN AUROC = 0.758
ROX Logistic Regression Prediction: MEAN AUROC = 0.487
ROX-HR Logistic Regression Prediction: MEAN AUROC = 0.480
