# Supplementary material for: Predicting Flow Rate Escalation for Pediatric Patients on High Flow Nasal Cannula Using Machine Learning
Source: Front Pediatr. 2021 Nov 8;9:734753. doi: 10.3389/fped.2021.734753 (PMC8606666; doi:10.3389/fped.2021.734753)
Supplement: Supplementary Figure 5 — Receiver operating characteristic curves comparing prediction models to baseline models for each lead time at a time window of 12 h. AUROCs for the logistic regression, random forest, and gradient boosting models are compared to baseline ROX logistic regression, ROX-HR logistic regression, and random prediction models. [file Image_5.PDF]

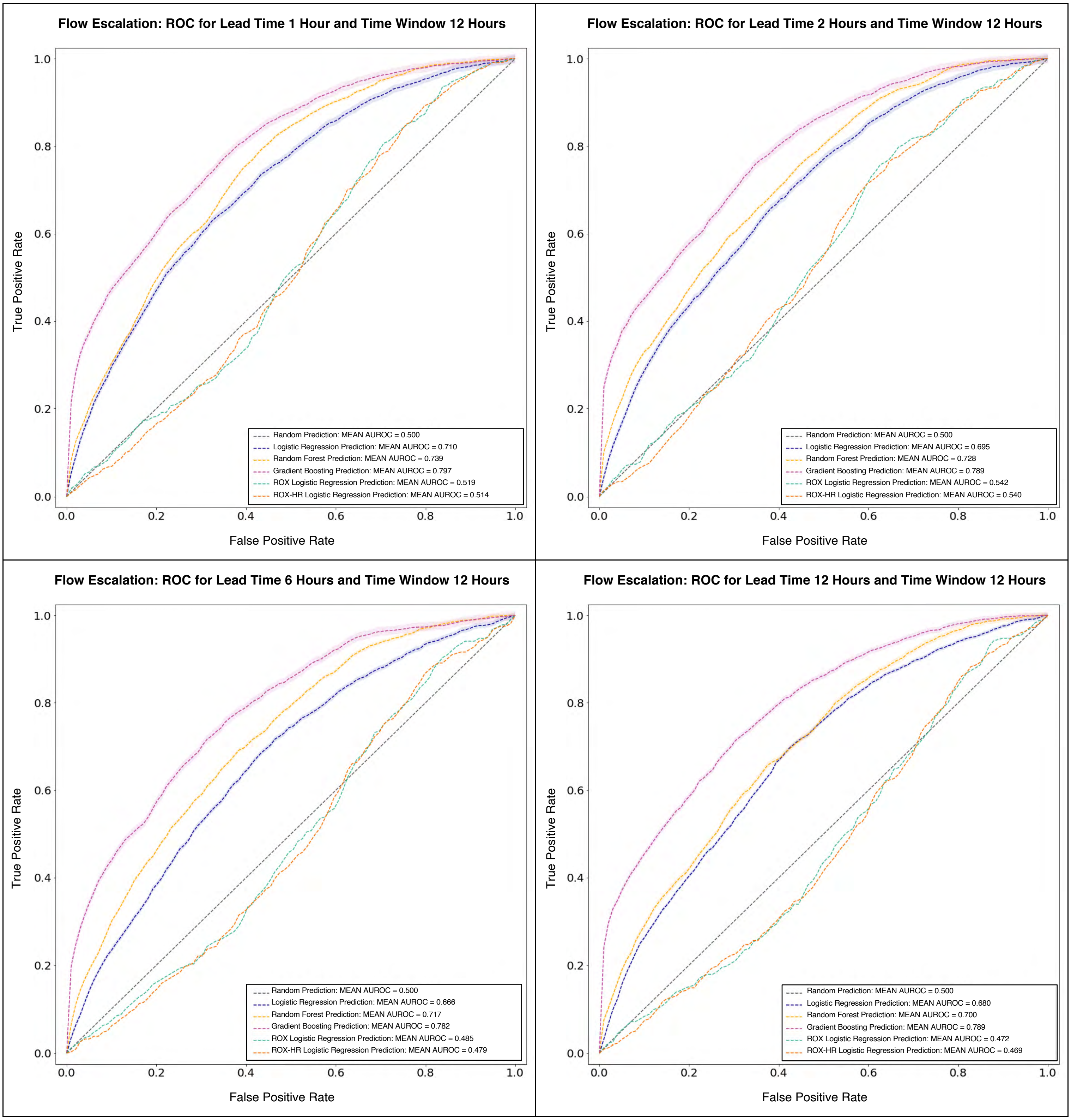

Flow Escalation: ROC for Lead Time 1 Hour and Time Window 12 Hours
True Positive Rate
False Positive Rate
Random Prediction: MEAN AUROC = 0.500
Logistic Regression Prediction: MEAN AUROC = 0.710
Random Forest Prediction: MEAN AUROC = 0.739
Gradient Boosting Prediction: MEAN AUROC = 0.797
ROX Logistic Regression Prediction: MEAN AUROC = 0.519
ROX-HR Logistic Regression Prediction: MEAN AUROC = 0.514
Flow Escalation: ROC for Lead Time 2 Hours and Time Window 12 Hours
True Positive Rate
False Positive Rate
Random Prediction: MEAN AUROC = 0.500
Logistic Regression Prediction: MEAN AUROC = 0.695
Random Forest Prediction: MEAN AUROC = 0.728
Gradient Boosting Prediction: MEAN AUROC = 0.789
ROX Logistic Regression Prediction: MEAN AUROC = 0.542
ROX-HR Logistic Regression Prediction: MEAN AUROC = 0.540
Flow Escalation: ROC for Lead Time 6 Hours and Time Window 12 Hours
True Positive Rate
False Positive Rate
Random Prediction: MEAN AUROC = 0.500
Logistic Regression Prediction: MEAN AUROC = 0.666
Random Forest Prediction: MEAN AUROC = 0.717
Gradient Boosting Prediction: MEAN AUROC = 0.782
ROX Logistic Regression Prediction: MEAN AUROC = 0.485
ROX-HR Logistic Regression Prediction: MEAN AUROC = 0.479
Flow Escalation: ROC for Lead Time 12 Hours and Time Window 12 Hours
True Positive Rate
False Positive Rate
Random Prediction: MEAN AUROC = 0.500
Logistic Regression Prediction: MEAN AUROC = 0.680
Random Forest Prediction: MEAN AUROC = 0.700
Gradient Boosting Prediction: MEAN AUROC = 0.789
ROX Logistic Regression Prediction: MEAN AUROC = 0.472
ROX-HR Logistic Regression Prediction: MEAN AUROC = 0.469
