# Supplementary material for: Predicting Flow Rate Escalation for Pediatric Patients on High Flow Nasal Cannula Using Machine Learning
Source: Front Pediatr. 2021 Nov 8;9:734753. doi: 10.3389/fped.2021.734753 (PMC8606666; doi:10.3389/fped.2021.734753)
Supplement: Supplementary Figure 6 — Shapley summary plot for our four synthetic features. Shapley additive explanations calculate the feature relevance for each individual prediction independently and depict it as a point in the figure. For each feature, this plot shows how a higher or lower value of that feature, represented by color, influences whether the model predicts a flow rate escalation (positive Shapley value) or not (negative Shapley value). The magnitude of the Shapley value indicates how strongly the model agrees with this prediction. [file Image_6.PDF]

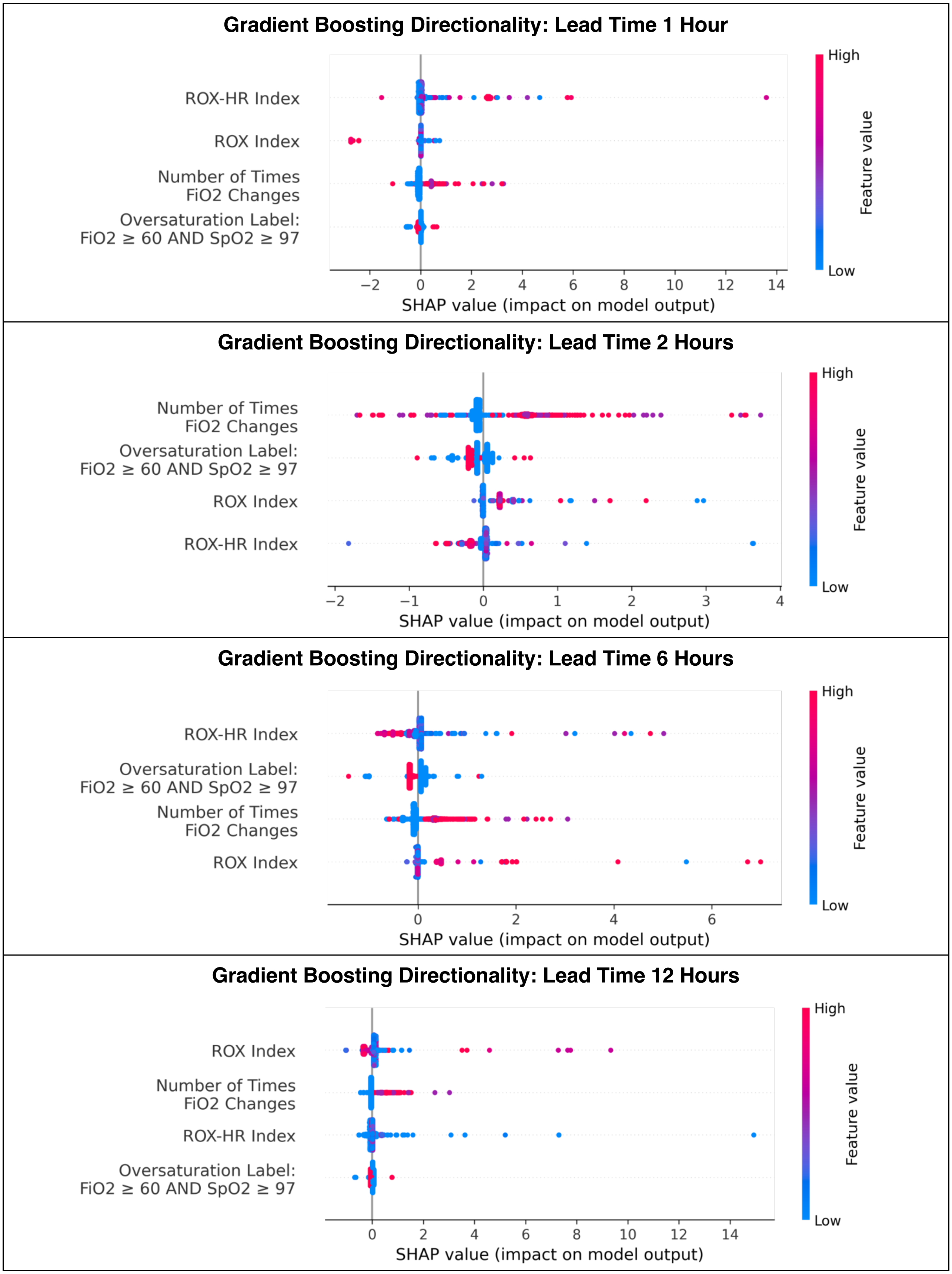

Gradient Boosting Directionality: Lead Time 1 Hour
ROX-HR Index
ROX Index
Number of Times FiO2 Changes
Oversaturation Label: FiO2 ≥ 60 AND SpO2 ≥ 97
−2
0
2
4
6
8
10
12
14
SHAP value (impact on model output)
High
Feature value
Low
Gradient Boosting Directionality: Lead Time 2 Hours
Number of Times FiO2 Changes
Oversaturation Label: FiO2 ≥ 60 AND SpO2 ≥ 97
ROX Index
ROX-HR Index
−2
−1
0
1
2
3
4
SHAP value (impact on model output)
High
Feature value
Low
Gradient Boosting Directionality: Lead Time 6 Hours
ROX-HR Index
Oversaturation Label: FiO2 ≥ 60 AND SpO2 ≥ 97
Number of Times FiO2 Changes
ROX Index
0
2
4
6
SHAP value (impact on model output)
High
Feature value
Low
Gradient Boosting Directionality: Lead Time 12 Hours
ROX Index
Number of Times FiO2 Changes
ROX-HR Index
Oversaturation Label: FiO2 ≥ 60 AND SpO2 ≥ 97
0
2
4
6
8
10
12
14
SHAP value (impact on model output)
High
Feature value
Low
