# Supplementary material for: Predicting Flow Rate Escalation for Pediatric Patients on High Flow Nasal Cannula Using Machine Learning
Source: Front Pediatr. 2021 Nov 8;9:734753. doi: 10.3389/fped.2021.734753 (PMC8606666; doi:10.3389/fped.2021.734753)
Supplement: Supplementary Table 1 — Average starting values of oxygen flow rate and fraction of inspired oxygen for each level of respiratory support. [file Table_1.docx]

**Supplementary Table 1.** Average starting values of oxygen flow rate and fraction of inspired oxygen for each level of respiratory support.

|  | **Room Air** | **Supplementary Oxygen** | **Regular Nasal Cannula** | **High Flow Nasal Cannula** | **Bilevel or Continuous Positive Airway Pressure** | **Conventional Mechanical Ventilation** |
| --- | --- | --- | --- | --- | --- | --- |
| Flow Rate (L/min) | 0.00 | 9.60 | 2.39 | 6.74 | --- | --- |
| F_i_O_2_ (%) | 21.00 | 42.20 | 88.87 | 40.81 | --- | --- |
