# Supplementary material for: Predicting Flow Rate Escalation for Pediatric Patients on High Flow Nasal Cannula Using Machine Learning
Source: Front Pediatr. 2021 Nov 8;9:734753. doi: 10.3389/fped.2021.734753 (PMC8606666; doi:10.3389/fped.2021.734753)
Supplement: Supplementary Table 2 — All of the features used in our model. [file Table_2.docx]

**Supplementary Table 2.** All of the features used in our model.

| Last Recorded F_i_O_2_ | Mean Respiratory Rate | Cephalosporin Antibiotics – 3rd Generation | Topical/Mucous Membr./Subcut. Enzymes | Metabolic Function Diagnostics | Phosphate Replacement |
| --- | --- | --- | --- | --- | --- |
| Number of Times F_i_O_2_ Changes | Change in Respiratory Rate | Absorbable Sulfonamide Antibacterial Agents | Emollients | Antifungal Agents | Pulmonary Anti-Htn., Endothelin Receptor Antagonist |
| Min F_i_O_2_ | Respiratory Rate Standard Deviation | Nutrition | Antilinteumdermatitis Agents, Topical | Antihistamines – 1st Generation | Eye Local Anesthetics |
| Max F_i_O_2_ | Number of Times Respiratory Rate Recorded | Vitamin D Preparations | Antihypertensives, Ace Inhibitors | Bile Salts | Vitamin C Preparations |
| Median F_i_O_2_ | Min SBP | Lincosamide Antibiotics | Anticonvulsants | Topical Anti-Inflammatory Steroidal | Antihistamines – 2nd Generation |
| Mean F_i_O_2_ | Max SBP | Penicillin Antibiotics | Mucolytics | Anticholinergics, Quaternary Ammonium | Acid And Alkali Poison Antidotes |
| Change in F_i_O_2_ | Median SBP | Glucocorticoids, Orally Inhaled | Anti-Anxiety - Benzodiazepines | Zink Supplement | Cefazolin |
| F_i_O_2_ Standard Deviation | Mean SBP | Beta-Adrenergic Agents, Inhaled, Continuous | Antihypertensives, Sympatholytic | Protectives | Bactrim |
| Number of Times F_i_O_2_ Recorded | Change in SBP | Electrolyte Maintenance | Pulm. Anti-Htn, Sel. C-Gmp Phosphodiesterase T5 Inhib. | Thrombolytic Enzymes | Amoxicillin |
| Min DBP | SBP Standard Deviation | Macrolide Antibiotics | Antifungal Antibiotics | Antiviral Monoclonal Antibodies | Ampicillin |
| Max DBP | Number of Times SBP Recorded | Nsaids, Cyclooxygenase Inhibitor - Type Analgesics | Pulmonary Antihypertensives, Prostacyclin-Type | Urinary Ph Modifiers | Cefepime |
| Median DBP | Min S_p_O_2_ | Topical Antibiotics | Topical Antifungals | Somatostatic Agents | Vancomycin |
| Mean DBP | Pediatric Vitamin Preparations | Glucocorticoids | Antivirals, General | Calcium Channel Blocking Agents | Augmentin |
| Change in DBP | Max S_p_O_2_ | Iron Replacement | Cephalosporin Antibiotics – 4th Generation | Topical Hemostatics | Azithromycin |
| DBP Standard Deviation | Median S_p_O_2_ | Thiazide And Related Diuretics | Adrenergic Agents, Catecholamines | Pineal Hormone Agents | Metronidazole |
| Number of Times DBP Recorded | Mean S_p_O_2_ | Aldosterone Antagonist, Potassium-Sparing Diuretic | Cephalosporin Antibiotics – 1st Generation | Topical Local Anesthetics | Clindamycin |
| Min MAP | Change in S_p_O_2_ | Laxatives, Local/Rectal | Selective Relaxant Binding Agents (SRBAS) | Metabolic Deficiency Agents | Age (Month) |
| Max MAP | S_p_O_2_ Standard Deviation | Antiflatulents | Gastrointestinal Radiopaque Diagnostics | General Bronchodilator Agents | Race: White (Non-Hispanic) |
| Median MAP | Number of Times S_p_O_2_ Recorded | Proton-Pump Inhibitors | Skeletal Muscle Relaxants | Platelet Aggregation Inhibitors | Race: Black |
| Mean MAP | Min Body Temperature | Potassium Replacement | Barbiturates | Anticonvulsant - Benzodiazepine Type | Race: Asian |
| Change in MAP | Max Body Temperature | Beta-Adrenergic And Anticholinergic Combo, Inhaled | Bicarbonate Producing/Containing Agents | Xanthines | Race: Unknown |
| MAP Standard Deviation | Median Body Temperature | Anaphylaxis Therapy Agents | Antiemetic/Antivertigo Agents | Beta-Adrenergic Blocking Agents | Race: Hispanic |
| Number of Times MAP Recorded | Mean Body Temperature | Magnesium Salts Replacement | Laxatives And Cathartics | IV Solutions: Dextrose and Lactated Ringers | Male |
| Min Pulse | Change in Body Temperature | Beta-Adrenergic Agents | Heparin And Related Preparations | OIP-Preventative Naloxone | Female |
| Max Pulse | Body Temperature Standard Deviation | Injection: Dextrose-Saline | Antifibrinolytic Agents | Immunosuppressives | Diagnosis at Admission: Problem Status Active |
| Median Pulse | Number of Times Body Temperature Recorded | Vitamin B1 Preparations | Vancomycin Antibiotics and Derivatives | Digitalis Glycosides | Diagnosis at Admission: Principal N |
| Mean Pulse | IV Solutions: Dextrose-Saline | Cardiovascular Diagnostics-Radiopaque | Opioid Infusion, PCA, Or Bolus | Antidiuretic And Vasopressor Hormones | Mean ROX Index |
| Change in Pulse | Histamine H2-Receptor Inhibitors | Mydriatics | Plasma Proteins | Artificial Tears | Mean ROX-HR Index |
| Pulse Standard Deviation | General Inhalation Agents | Vitamin K Preparations | Calcium Replacement | Nose Preparations, Vasoconstrictors (OTC) | Oversaturation Label: F_i_O_2_≥60 & S_p_O_2_≥97 |
| Number of Times Pulse Recorded | Sodium/Saline Preparations | Ophthalmic Antibiotics | Opioid Analgesics | Diagnostic Preparations, Misc. | *Abbreviations  DBP = Diastolic Blood Pressure  SBP = Systolic Blood Pressure  MAP = Mean Arterial Pressure  PCA = Patient-controlled Analgesia  OTC = Over-the-counter |
| Min Respiratory Rate | Analgesic/Antipyretics, Non-Salicylate | IV Solutions: Dextrose-Water | Sympathomimetic Agents | Anticholinergics, Orally Inhaled Short Acting |  |
| Max Respiratory Rate | Intestinal Motility Stimulants | IV Fat Emulsions | Inotropic Agents | Leukocyte (WBC) Stimulants |  |
| Median Respiratory Rate | Loop Diuretics | Nose Preparations, Misc. (OTC) | Coagulants | Fibrinolytic Agents |  |
