# Supplementary material for: Predicting Flow Rate Escalation for Pediatric Patients on High Flow Nasal Cannula Using Machine Learning
Source: Front Pediatr. 2021 Nov 8;9:734753. doi: 10.3389/fped.2021.734753 (PMC8606666; doi:10.3389/fped.2021.734753)
Supplement: Supplementary Table 3 — Performance metrics comparing gradient boosting, ROX, and ROX-HR model performances to a random classifier at a time window of 8 h. The metrics include sensitivity, specificity, mean AUROC, mean AUPRC, positive predictive value (PPV), and negative predictive value (NPV). The PPV for ROX and ROX-HR logistic regression models could not be obtained, because these models predicted no true positive and false positive values. [file Table_3.docx]

**Supplementary Table 3.** Performance metrics comparing gradient boosting, ROX, and ROX-HR model performances to a random classifier at a time window of 8 hours. The metrics include sensitivity, specificity, mean AUROC, mean AUPRC, positive predictive value (PPV), and negative predictive value (NPV). The PPV for ROX and ROX-HR could not be obtained, because these models predicted no true positive and false positive values.

| **Model Name** | **Sensitivity** | **Specificity** | **AUROC** | **AUPRC** | **PPV** | **NPV** |
| --- | --- | --- | --- | --- | --- | --- |
| Random, No-Skill  Lead Time 1 hr.  Lead Time 2 hrs.  Lead Time 6 hrs.  Lead Time 12 hrs. | 0.500 ± 0.000  0.500 ± 0.000  0.500 ± 0.000  0.500 ± 0.000 | 0.500 ± 0.000  0.500 ± 0.000  0.500 ± 0.000  0.500 ± 0.000 | 0.500 ± 0.000  0.500 ± 0.000  0.500 ± 0.000  0.500 ± 0.000 | 0.020 ± 0.000  0.020 ± 0.000  0.020 ± 0.000  0.020 ± 0.000 | 0.500 ± 0.000  0.500 ± 0.000  0.500 ± 0.000  0.500 ± 0.000 | 0.500 ± 0.000  0.500 ± 0.000  0.500 ± 0.000  0.500 ± 0.000 |
| Gradient Boosting  Lead Time 1 hr.  Lead Time 2 hrs.  Lead Time 6 hrs.  Lead Time 12 hrs. | 0.728 ± 0.060  0.728 ± 0.067  0.694 ± 0.062  0.686 ± 0.065 | 0.727 ± 0.081  0.687 ± 0.081  0.687 ± 0.081  0.677 ± 0.081 | 0.810 ± 0.003  0.778 ± 0.007  0.775 ± 0.009  0.758 ± 0.012 | 0.153 ± 0.028  0.081 ± 0.009  0.192 ± 0.038  0.105 ± 0.010 | 0.476 ± 0.169  0.264 ± 0.064  0.634 ± 0.215  0.287 ± 0.066 | 0.986 ± 0.000  0.988 ± 0.000  0.985 ± 0.000  0.988 ± 0.000 |
| ROX Logistic Regression  Lead Time 1 hr.  Lead Time 2 hrs.  Lead Time 6 hrs.  Lead Time 12 hrs. | 0.675 ± 0.096  0.563 ± 0.094  0.568 ± 0.088  0.506 ± 0.088 | 0.414 ± 0.081  0.455 ± 0.081  0.495 ± 0.081  0.475 ± 0.081 | 0.525 ± 0.000  0.506 ± 0.000  0.537 ± 0.000  0.487 ± 0.000 | 0.017 ± 0.000  0.019 ± 0.000  0.015 ± 0.000  0.015 ± 0.000 | ---  ---  ---  --- | 0.985 ± 0.000  0.987 ± 0.000  0.983 ± 0.000  0.988 ± 0.000 |
| ROX-HR Logistic Regression  Lead Time 1 hr.  Lead Time 2 hrs.  Lead Time 6 hrs.  Lead Time 12 hrs. | 0.617 ± 0.094  0.639 ± 0.093  0.620 ± 0.089  0.629 ± 0.089 | 0.455 ± 0.081  0.404 ± 0.081  0.465 ± 0.081  0.374 ± 0.081 | 0.525 ± 0.000  0.502 ± 0.000  0.536 ± 0.000  0.480 ± 0.000 | 0.016 ± 0.000  0.017 ± 0.000  0.015 ± 0.000  0.015 ± 0.000 | ---  ---  ---  --- | 0.985 ± 0.000  0.987 ± 0.000  0.983 ± 0.000  0.988 ± 0.000 |
